# Do Saudi medical schools consider the core topics in undergraduate medical curricula?

**DOI:** 10.1186/s12909-022-03452-1

**Published:** 2022-05-17

**Authors:** Amro K. Bin Abdulrahman, Abdulrahman Yousef Aldayel, Khalid A. Bin Abdulrahman, Yousef Rafat Bukhari, Yazeed Almotairy, Saleh Aloyouny, Hamad Qabha, Mansour Almadi, Mohammed Almasri, Abdulaziz Alasmari, Abdullah Alghamdi, Yasir Alotaibi, Abdulmajeed Bin Dahmash, Muteb Mousa Alharbi, Asem M. Shadid

**Affiliations:** 1 Preventive Medicine, Department of Public Health, Ministry of Health, Riyadh, Saudi Arabia; 2 Prince Sultan Military Medical City, Riyadh, Saudi Arabia; 3 Department of Medical Education, College of Medicine, Imam Mohammad Ibn Saud Islamic University (IMSIU), Othman Bin Affan Rd. Al-Nada, Riyadh, 7544 Saudi Arabia; 4 King Faisal Specialist Hospital & Research Centre, Riyadh, Saudi Arabia; 5 Department of Internal Medicne, Ministry of National Guard Health Affairs, Riyadh, Saudi Arabia; 6 Department of Radiology, Dallah Hospitals, Riyadh, Saudi Arabia; 7 Department of Dermatology, King Fahad Medical City, Riyadh, Saudi Arabia

**Keywords:** Saudi Arabia, Core topics, Undergraduate curriculum, Medical education

## Abstract

**Background:**

Most of the medical schools in Saudi Arabia are currently evolving their curricula in accordance with the most recognized medical education trends worldwide. Undergraduate medical school’s curriculum should be compatible with community health needs. Therefore, the study aims to explore the current contents of Saudi undergraduate medical curricula and to check if the core topics that were internationally recognized were implemented in their curriculum.

**Methodology:**

An online questionnaire was designed and sent to 37 deans of medical schools in Saudi Arabia. The deans or the vice-deans in charge of the curriculum were asked to complete the pre-designed questionnaire, which assessed the status of inclusion of the core topics in the curriculum of their affiliated schools. Each listed core topic was evaluated according to five options for each subject: not included, separate required course, part of the required course, separate elective course, and part of an elective course.

**Results:**

Twenty four out of 37 (65%) Saudi medical schools completed the survey questionnaire. Almost all core topics, such as communication skills, evidence-based medicine, patient safety, professionalism were included in the curricula of Saudi medical schools as separate required courses or as part of required courses or elective courses.

Complementary and alternative medicine and the history of medicine were the topics least taught in Saudi medical colleges, as 25% of the schools did not include them in their curricula.

**Conclusion:**

The majority (65%) of the internationally recognized core topics were included in the Saudi undergraduate medical curricula. Evidence-based medicine, complementary medicine, the Saudi healthcare system, patient safety, and professionalism/medical ethics should be part of compulsory credited courses in all Saudi undergraduate medical curricula.

## Introduction

In the last two decades, Saudi medical colleges have witnessed rapid development in medical education. Perhaps one of the most prominent reasons for this is the significant expansion in opening new medical colleges, which made medical education leaders urge the administration of emerging colleges to adopt modern curricula and learning strategies adopted by most international medical colleges [1, 2]. The Saudi Deans Committee and the Saudi Society for Medical Education had a significant role in adopting modern trends in medical education. This was represented in launching the initiative of the national framework for the competencies of graduates of Saudi medical colleges [3]. Currently, Saudi Arabia has 28 public and nine private medical schools [4]. Recently, new and different courses have been implemented which did not exist before. These new topics have been implemented in the Saudi undergraduate medical curricula. Most old medical schools in Saudi Arabia have already adopted integrated, problem-based, community-oriented curricula [5]. However, an undergraduate medical school’s curriculum should be compatible with community health needs [6].

The Saudi Medical Deans’ Committee founded the Saudi MEDs initiative to establish expected core learning outcomes (LOs)/competencies for medical degree programs in Saudi Arabia. It aimed to harmonize the Saudi Medical Higher Education Sector [3]. The SaudiMEDS framework has great potential to improve the quality of Saudi medical graduates to meet current and future needs [7]. Medical curriculum planners should ensure that core topics such as professionalism, communication skills, evidence-based practice, patient safety, healthcare systems, research methods, and other essential topics are part of the undergraduate medical curriculum [8, 9].

The current study aimed to examine whether Saudi undergraduate medical schools include the most internationally recognized core topics in their undergraduate medical curricula.

## Methodology

The researchers conducted a website search to look at the medical programs of 15 randomly selected medical schools representing North America, Europe, the Middle East, Asia, and Australia (three medical schools representing each continent). The purpose of the preset search was to look for the common core topics in the undergraduate medical curricula. The core topics were defined as those that a medical student cannot graduate from the college without understanding its basics, how to apply them, and their ethical aspects of medical practice. The typical example of core topics are medical ethics, patient safety, and evidence-based medical practice. Medical essential courses that are part of the study of medicine in all medical schools, such as applied anatomy, medical physiology, pharmacology, surgery, internal medicine, pediatrics, obstetrics and gynecology, family medicine, and other basic and clinical medical courses were excluded. A form was designed containing the core topics that fit the defined criteria. The research team reviewed and audited the list in three consecutive phases. The first phase, was the inclusion and exclusion stage. The second phase was the filtration and classification of core topics. Whereas, the third phase was final auditing and approving the list of core topics (Table 1). After that, a survey questionnaire was designed to examine the presence of the international core topics in the Saudi medical curricula. The survey was subjected to a pilot testing on 25 volunteered faculty members at Imam Mohammad Ibn Saud Islamic University, the survey was modified accordingly. The survey was distributed using SurveyMonkey to the email addresses and mobile numbers of all 37 deans of public and private medical schools in Saudi Arabia [10]. The deans or the vice-deans in charge of the curriculum were asked to complete the online survey, which examined the status of the core topics in the undergraduate medical curriculum of each Saudi school. It was clearly explained to the deans and vice-deans about the objectives of the survey, the risks and benefits from participating in this research. They were told that all their responses will be treated with confidentiality and it’s only used for research purposes. Each listed core topic was assessed according to five options for each subject: not included, separate required course, part of the required course, separate elective course, and part of the elective course. Three follow-up reminder messages were sent, one each after two days, five days, and seven days had passed since the initial message. The survey proposal was approved by the IRB committee of Imam Mohammad Ibn Saud Islamic University (HAPO-01-R-011) session 38–2017 dated October 20, 2017. All participants have taken the consent form before filling the survey.

Data were analyzed using the Statistical Package for the Social Sciences (IBM, Armonk, NY, USA) software, version 20. The frequency and proportions were used to describe the categorical variables. Excel was used for creating figures and depictions.

## Result

**Fig. 1:**
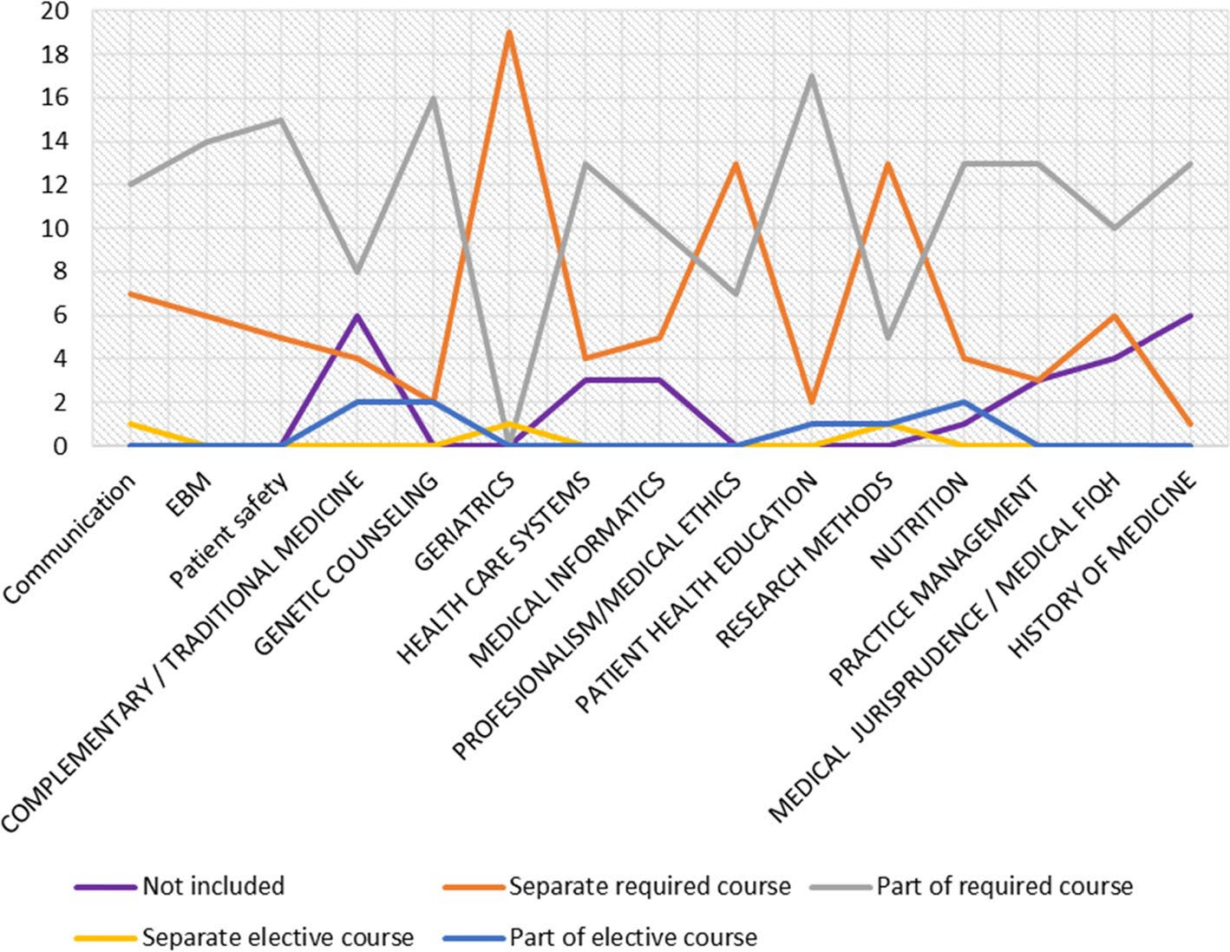
Distribution of responses towards the core topics in the curriculum

The majority 24 (65%) of the studied Saudi medical schools completed the survey questionnaire. Almost all essential core topics such as communication skills, evidence-based practice, patient safety, professionalism, genetic counseling, geriatrics, patient health education, and research methods were included in Saudi undergraduate medical curricula as separate required courses or as part of required courses or elective courses. The majority of Saudi medical curricula considered professionalism/medical ethics and research methods as separate required courses. Although some important topics (such as evidence-based practice, patient safety, genetic counseling, healthcare systems, patient health education, nutrition therapy, practice management, and the history of medicine) deserve to be separate required courses, the majority (67–84%) consider them to be part of required courses (Table 2). Complementary/traditional medicine and the history of medicine were found to be the least-taught topics in Saudi medical colleges, as 25% of the schools do not include them in their curricula. Medical jurisprudence/medical fiqh was the second least-implemented subject in Saudi medical colleges, as 17% of the schools do not include them in their curricula. After that, healthcare systems, medical informatics, and practice management were considered the third least-implemented topics, as 13% of the schools do not include them in their curricula (Table 2 and Fig. 1).

**Table 1:** The reference list of core topics in the international medical schools *n* = 15

	**Core Topics**	**Frequency %**^**a**^
1	Communication skills	14 (93%)
2	Professional/Medical ethics	13 (87%)
3	Evidence-based practice	12 (87%)
4	Patient safety	12 (83%)
5	Geriatrics	11 (73%)
6	Research methods	11 (73%)
7	Genetic counseling	10 (67%)
8	Patient health education	10 (67%)
9	Nutrition therapy	9 (60%)
10	Medical informatics	8 (53%)
11	Complementary/Traditional medicine	8 (53%)
12	Healthcare systems	8 (53%)
13	Practice management	6 (40%)
14	Medical jurisprudence/Medical fiqh/ Medical law	2 (13%)
15	History of medicine	2 (13%)

^a^The percentage of commonality included in the 15 international medical schools

**Table 2:** Distribution of responses towards the core topics in Saudi medical curricula (*n* = 24)

Core Topics	Not included n(%)	Separate required course n(%)	Part of required course n(%)	Separate elective course n(%)	Part of elective course n(%)
Communication skills	0(0%)	9 (37%)	14 (59%)	1 (5%)	0(0%)
Evidence-based practice	0(0%)	8(33%)	16(67%)	0(0%)	0(0%)
Patient safety	0(0%)	7(29%)	17(71%)	0(0%)	0(0%)
Complementary/Traditional medicine	6(25%)	6(25%)	10(42%)	0(0%)	2(8%)
Genetic counseling	0(0%)	2(8%)	20(84%)	0(0%)	2(8%)
Geriatrics	0(0%)	2(8%)	14(59%)	8(33%)	0(0%)
Healthcare systems	3(13%)	4(17%)	17(70%)	0(0)	0(0)
Medical informatics	3(13%)	5(21%)	12(50%)	0(0)	4(16%)
Professional/Medical ethics	0(0%)	16(67%)	8(33%)	0(0%)	0(0%)
Patient health education	0(0%)	2(8%)	20(84%)	0(0%)	2(8%)
Research methods	0(0%)	17(71%)	5(21%)	1(4%)	1(4%)
Nutrition therapy	1(4%)	4(17%)	17(71%)	0(0)	2(8%)
Practice management	3(12.5%)	3(12.5%)	18(75%)	0(0)	0(0)
Medical jurisprudence/Medical fiqh	4(17%)	7(29%)	13(54%)	0(0)	0(0)
History of medicine	6(25%)	1(4%)	17(71%)	0(0)	0(0)

## Discussion

To our knowledge, this is the first study conducted in Saudi Arabia that addressed this area in medical education. Almost all essential core topics were included in Saudi undergraduate medical curricula as separate required courses or as part of required courses or elective courses. This finding was supported by a previous study undertaken in 30 medical schools in Gulf cooperative countries [5]. The current study found that most medical schools teach evidence-based medicine either as a separate course or as a part of other courses. Likewise, most medical schools in the United States and Canada incorporate evidence-based medicine (EBM) in their curricula [11]. However, our findings showed that only 33% of medical colleges in Saudi Arabia teach EBM as a core course.

Meanwhile, in the United Kingdom, most medical schools teach EBM as a separate course [12]. Communication skills were a component of all Saudi medical curricula. Because communication is an essential skill for any medical professional, this was clearly emphasized in the SaudiMEDs Framework initiative [3, 7]. Moreover, communicative training is one of the primary educational tasks of higher medical education in the United States. Therefore, communication skills courses are a necessary component of medical curricula throughout all years of study [13]_._

Furthermore, clinical communication skills teaching and learning form a core part of the modern undergraduate medical curriculum in most medical schools in the United Kingdom [14]. Therefore, communication skills provide many benefits if they are correctly applied in the field of medicine. For example, communication skills lead to better patient treatment outcomes, help one understand and influence patients’ physical and psychological health, enhance treatment and physician recommendations to patients, and ultimately improve the working atmosphere and job satisfaction [14]. All this makes it logical to teach communication skills in all medical schools in Saudi Arabia.

Professionalism and medical ethics is a crucial course in medical education. Students should learn moral behaviors and professionalism so that they can become better physicians. In the medical field and hospitals, physicians encounter a variety of ethical dilemmas. These dilemmas are inevitable and should be handled correctly. Adding ethics to medical curricula is necessary so that students can handle such difficulties in the future. This course enlightens students with the general rules of morality that they can use to make decisions. Most medical schools in the United Kingdom teach medical ethics in their undergraduate curricula. A study performed at the University of Glasgow Medical School in England stated that students showed improvement after their first year of learning ethics in small groups and that their answers were similar [15]. Almost all Saudi medical schools were teaching professionalism and medical ethics as separate required courses.

In the past decade, the teaching of patient safety to trainees in the United States has increased. The American Association of Medical Colleges (AAMC) has included this course in its postgraduate medical curriculum because a significant increase in annual medical errors (90,000) has been occurring in the United States [16]. Saudi Arabia has established this course in most of its medical curricula*.*

Most medical schools in different countries consider management and leadership skills essential in medical education. In the United States, 46 (54.5%) out of 88 medical schools reported exposure to leadership skills within their undergraduate medical education as either a required course or an elective course, or as both [17, 18].

It is not surprising to observe that the majority (83%) of Saudi medical curricula include medical jurisprudence/medical fiqh in undergraduate medical programs. This is because Saudi Arabian people are Muslims and most Saudi patients are keen to apply Islamic law (fiqh) to health-related practices.

Patient education is a significant component of modern healthcare [19]. The goals of health education are to change and promote societal health behaviors. In Saudi Arabia, medical schools have understood this issue, as 84% of medical schools have added patient health education as a required course. In comparison, 8% include it as a separate required course. This may be because many Gulf Cooperation Council countries – including Saudi Arabia – saw remarkable growth in their healthcare systems between the 1970s and 1990s [20]. Since then, the government of Saudi Arabia has made an effort to improve healthcare through patient health education. This remains a focal point of healthcare policies today.

Out of 114 medical schools in the United States and Canada, only two didn’t teach genetic counseling. Of the remaining 112 schools that did teach genetic counseling, 52 taught it as a separate course, and 60 taught it as part of other courses [21]. The authors believe that the interest in genetic counseling in Saudi medical schools is due to the increased risk of congenital disease, possibly explained by the increasing amount of consanguinity stemming from the Saudi Arabian tradition. Thus, premarital and prenatal genetic counseling and testing are of increased importance for detecting genetic diseases.

Our results showed that the majority of Saudi medical schools are implementing research methodology courses in their undergraduate curricula. Unfortunately, some students do not appreciate the importance of acquiring research methods skills as early as the pre-clinical years of their undergraduate education. This leads to many studies conducted using inappropriately described methodologies and a lack of knowledge regarding statistical methods and their misuse. In response, there have been calls to implement research methods courses with sufficient statistical training in biomedical education [22, 23].

Our finding matches those of other studies [24–26], which encourages the addition of undergraduate-level courses that may offer many benefits for students, faculty, universities, and even the country, especially in terms of publishing excellent papers.

## Conclusion

The Saudi undergraduate medical curricula contained a majority (65%) of internationally recognized core topics. Evidence-based medicine, complementary medicine, the national health care system, patient safety, and professionalism/medical ethics were not considered as a separate course, and some were not included at all. Therefore, they should be part of compulsory credited courses in all Saudi undergraduate medical curricula. Saudi deans committee should call for an initiative to ensure that all core topics should be part of required courses in all Saudi medical schools’ curricula.

## Data Availability

The datasets analyzed during the current study is available from the corresponding author on reasonable request. Due to data protection restrictions and participant confidentiality, we do not make participant data publicly available.
